# Host range and molecular and ultrastructural analyses of Asparagus virus 1 pathotypes isolated from garden asparagus *Asparagus officinalis* L.

**DOI:** 10.3389/fpls.2023.1187563

**Published:** 2023-07-31

**Authors:** Edit Lantos, Reiner Krämer, Katja R. Richert-Pöggeler, Edgar Maiss, Janine König, Thomas Nothnagel

**Affiliations:** ^1^Julius Kühn-Institut, Federal Research Centre of Cultivated Plants, Institute of Breeding Research on Horticultural Crops, Quedlinburg, Germany; ^2^Julius Kühn-Institut, Federal Research Centre of Cultivated Plants, Institute for Epidemiology and Pathogen Diagnostics, Braunschweig, Germany; ^3^Leibniz-University Hannover, Institute of Horticultural Production Systems, Hannover, Germany

**Keywords:** *Asparagus officinalis*, Asparagus virus 1 (AV1), virus incidence, host range, pathotype, ultrastructural changes, cylindrical inclusions, phylogeny of coat protein region

## Abstract

Asparagus samples were examined from growing areas of Germany and selected European as well as North, Central and South American countries. Overall, 474 samples were analyzed for Asparagus virus 1 (AV1) using DAS-ELISA. In our survey, 19 AV1 isolates were further characterized. Experimental transmission to 11 species belonging to Aizoaceae, Amarantaceae, Asparagaceae, and Solanaceae succeeded. The ultrastructure of AV1 infection in asparagus has been revealed and has been compared with the one in indicator plants. The cylindrical inclusion (CI) protein, a core factor in viral replication, localized within the cytoplasm and in systemic infections adjacent to the plasmodesmata. The majority of isolates referred to pathotype I (PI). These triggered a hypersensitive resistance in inoculated leaves of *Chenopodium* spp. and were incapable of infecting *Nicotiana* spp. Only pathotype II (PII) and pathotype III (PIII) infected *Nicotiana benthamiana* systemically but differed in their virulence when transmitted to *Chenopodium* spp. The newly identified PIII generated amorphous inclusion bodies and degraded chloroplasts during systemic infection but not in local lesions of infected *Chenopodium* spp. PIII probably evolved *via* recombination in asparagus carrying a mixed infection by PI and PII. Phylogeny of the coat protein region recognized two clusters, which did not overlap with the CI-associated grouping of pathotypes. These results provide evidence for ongoing modular evolution of AV1.

## Introduction

Garden asparagus (*Asparagus officinalis* L.) out of the family Asparagaceae is a perennial vegetable. In Germany, asparagus is a favored seasonal crop, whose young white or green spears are harvested and consumed during springtime. During cultivation and its whole life span, asparagus is exposed to a number of pathogens, particularly fungi and viruses (Evans et al., 1990; Kegler et al., 1991; Elmer et al., 1996; Nothnagel et al., 2011; Tomassoli et al., 2012; Elmer, 2018). Asparagus virus 1 (AV1) causing economic losses was first described in Germany (Hein, 1960). AV1 is grouped into the genus *Potyvirus*, family *Potyviridae* based on virion morphology, genome organization, and its nonpersistent transmission by the aphid vectors *Myzus persicae* and *Aphis craccivora* (Fujisawa et al., 1983). Among the *Potyvirus*-encoded proteins, the cylindrical inclusion (CI) protein is a core factor in viral replication and virulence (Sorel et al., 2014). Thus, it displays multiple functions and interacts with various proteins both of viral and host origin during viral infection (Revers and Garcia, 2015). The CI protein acts as an ATP-dependent helicase essential for RNA synthesis. As indicated by the name, the CI forms visible inclusions aka “pinwheels” (pw) in infected cells that are important for plasmodesmata targeting and intercellular transport of replication vesicles through the plasmodesmata (Movahed et al., 2017).

AV1 has a restricted host range, with asparagus as the only known natural host (Brunt et al., 1996). AV1 is endemic in all asparagus-growing countries worldwide. Previous investigations reported an AV1 infection status of 90% up to 100% in asparagus fields in different countries (Howell and Mink, 1985; Montasser and Davis, 1987; Kegler et al., 1991; Bandte et al., 2008; Knaflewski et al., 2008; Tomassoli et al., 2008; Nothnagel et al., 2013). Even though AV1-infected phylloclades do not show a symptomatic phenotype, plant vigor and resilience are affected. Economically significant reductions in yield and quality of asparagus spears as well as an increasing susceptibility of asparagus plants to *Fusarium* spp. have been observed (Weissenfels and Schmelzer, 1976; Yang, 1979; Evans et al., 1989; Kegler et al., 1991; Tiberini et al., 2014; Lantos et al., 2018). In greenhouse experiments, AV1 infection reduced the shoot length (12%–20%), the shoot weight (10%–33%), the total root area (18%–47%), and the total root weight (31%–61%), as well as changes in volatile organic compounds in asparagus cultivars infected with AV1 have been demonstrated (Lantos et al., 2018). Earlier studies showed that mixed infections of AV1, together with asparagus virus 2 (AV2) and/or cucumber mosaic virus (CMV), cause yield reduction between 30% and 70% (Hein, 1963; Weissenfels and Schmelzer, 1976; Yang, 1979; Evans et al., 1990; Kegler et al., 1991; De Vries-Paterson et al., 1992; Jaspers et al., 1999; Kegler et al., 1999; Fiedorow et al., 2001). Thus, AV1 also contributes to the asparagus decline syndrome (Elmer et al., 1996; Knaflewski et al., 2008; Elmer, 2018). An effective control of the AV1 requires asparagus cultivars resistant to AV1. However, no AV1-resistant asparagus cultivars are currently available (Kegler et al., 1999; Knaflewski et al., 2008; Nothnagel et al., 2013; Nothnagel et al., 2014; Nothnagel et al., 2017). Extensive research identified AV1-resistant genotypes in 29 wild relatives of asparagus, which serve as a putative resource for introgression breeding programs (Nothnagel et al., 2017; Plath et al., 2018). For a durable AV1 resistance in garden asparagus, it is essential to determine the diversity of existing AV1 isolates and to characterize their biological properties. Until now, only few attempts were made to analyze the variability of AV1 isolates (Tomassoli et al., 2007; Tomassoli et al., 2008). The phylogenies of 19 AV1 isolates from Italy, Germany, United States, Peru, and Mexico using partial nucleotide sequences of the coat protein (CP/3’UTR) region were investigated by Tiberini et al. (Tiberini et al., 2014). The obtained phylogenetic tree showed two clusters (A and B) with very low variability within branches. Among the German AV1 isolates, two pathotypes have been identified that differed in their ability to infect *Nicotiana benthamiana* (Rabenstein et al., 2007). On the molecular level, 37 non-synonymous nucleotide substitutions, resulting in 15 amino acid sequence differences, spanning the whole coding region were identified between them (Blockus et al., 2015).

The objective of this study was to investigate the incidence of AV1 in the asparagus-growing regions of Germany and selected countries, to examine the pathogenicity of isolates, and to establish a collection of AV1 isolates distinct for their virulence. The latter are intended for further use in resistance screenings during breeding programs. We performed comprehensive analyses covering host range, virulence, pathogenesis, and genetic diversity of AV1 isolates. The impact of the studied parameters to improve strategies of AV1 control is discussed.

## Materials and methods

### Verification of AV1 in asparagus samples

Asparagus phylloclades originated from commercial asparagus fields throughout Germany’s growing regions, from selected European countries and North America, as well as asparagus spears from supermarkets (Greece, Hungary, Mexico, Peru, and Spain), in total 474 samples, were examined for infection with AV1 (**Table 1**). The presence of AV1 was tested by double-antibody sandwich enzyme-linked immunosorbent assay (DAS-ELISA) following a protocol of Clark and Adams (1977) in each sample. The polyclonal antibodies for AV1 originated from the serum bank of the JKI provided by Dr. Heiko Ziebell, JKI-EP. For AV1 detection, shoot branches of approximately 10 cm from the lower, middle, and upper part of an asparagus plant, including phylloclades, were harvested. The spear samples from supermarkets were cut into small pieces. From each plant, a mixed sample of 300 mg (phylloclade or spear tissue) was weighted and 3-ml extraction buffer PBST (phosphate buffered saline, Sigma-Aldrich, with Tween + 2% polyvinylpyrrolidone) was added according to a protocol of the German Collection of Microorganisms and Cell Cultures (DSMZ, Braunschweig, Germany). Samples were homogenized using a HOMEX Homogenizer and extraction bags (Bioreba, Reinach, Switzerland). The absorbance was measured with the microplate reader MRX II (Tecan Group Ltd., Switzerland) at 405 nm. Only isolates that were AV1 positive and did not show mixed infections with CMV and AV2 were chosen (compare **Table 1**).

**Table 1:** List of tested samples originated from different asparagus-growing regions.

Country origin	Location	Samples (n)	Cultivar	Plant age	AV1 (n) %	Code (GenBank no.)
Europe						
Germany						
Baden-Württemberg	Nordbaden	20	Gijnlim	3	(20) 100	AV1/16 (ON548333)
	Freiburg	20	Backlim	3	(20) 100	
Bavaria	Franken	20	Gijnlim	4	(20) 100	AV1/9 (ON548331)
	Niedermotzing	20	Backlim	3	(20) 100	
Brandenburg	Klaistow	20	Gijnlim	3	(20) 100	AV1/14 (ON548326)
Hesse	South Hesse	20	Gijnlim	4	(20) 100	AV1/19 (ON548337)
Lower Saxony	Lower Saxony east	20	Backlim	3	(20) 100	AV1/7 (ON548329)
	Lower Saxony west	20	Grolim	4	(20) 100	
North Rhine-Westphalia	Sendenhorst	20	Gijnlim	4	(20) 100	AV1/10 (ON548332)
Rhineland-Palatinate	–	20	Gijnlim	4	(19) 95	
	Rhineland	20	Cumulus	4	(20) 100	
	–	19	Backlim	3	(19) 100	AV1/8 (ON548330)
	Ingelheim	5	Gijnlim	3	(5) 100	–
	Ingelheim	5	Backlim	3	(5) 100	
	Ingelheim	5	Cumulus	3	(5) 100	
Saxony-Anhalt	Lindau	5	Ramada	9	(5) 100	–
	Lindau	1	Primaverde	6	(1) 100	
	Lindau	7	Ravel	6	(7) 100	
	Lindau	2	Ramires	2	(2) 100	
	Tangerhütte	20	Backlim	4	(20) 100	
	Möringen	1	–	–	(1) 100	AV1/1 (ON548319)
	Quedlinburg	1	–	–	(1) 100	AV1/2 (ON548323)
	Aschersleben	1	–	–	(1) 100	AV1/5 (ON548320)
Schleswig-Holstein	Wiemersdorf	20	Ramires	4	(20) 100	AV1/15 (ON548327)
**Austria**	Lower Austria	20	Vitalim	4	(20) 100	AV1/17 (ON548334)
**Netherlands**	Neer	20	Herkolim	4	(20) 100	AV1/18 (ON548335)
**Greece**	Galatades	5*	–	–	(5) 100	AV1/6 (ON548328)
**Hungary**	Kiskőrös	4*	–	–	0	
**Spain**	Granada	8*	–	–	(2) 25	
North and South America						
Canada	Ontario	20	Guelph Millennium	4	(20) 100	
United States	Michigan	5	Guelph Millennium	3	(5) 100	
	Michigan	5	Challenger 2	3	(5) 100	
	Michigan	5	NJ1113	3	(4) 80	AV1/20 (ON548336)
	Michigan	5	Jersey Night	3	(5) 100	
Mexico	–	15*	–	–	(2) 13.3	
Peru	Trujillo	53*	–	–	(17) 32.1	
Provided isolates						
JKI	Rastatt	1	–	–	(1) 100	AV1/4 (ON548322)
DSMZ^1^ PV-0955	Möringen	1	–	–	(1) 100	AV1/11 (ON548324)
DSMZ^2^ PV-0954	Rastatt	1	–	–	(1) 100	AV1/12 (ON548321)
Nunhems^3^	–	1	–	–	(1) 100	AV1/13 (ON548325)

^1^PV-0955, origin: Rastatt, Baden-Württemberg, Germany. ^2^PV-0954, origin: Möringen, Saxony-Anhalt, provided by the German Collection of Microorganisms and Cell Cultures DSMZ (Braunschweig, Germany). ^3^Nunhems Netherlands B.V. *Spears from supermarket. AV1 (n)% - ratio and percent of AV1-infested plants from Sample (n).

(-) Sample/Cultivar: unknown, (-) Code: AV1 not isolated because mixed infection with CMV or/and AV2.

### Experimental host range and determination of pathotypes

Plants used for the experimental host range study were selected based on previous literature data (Hein, 1960; Hein, 1969; Gröschel, 1976; Weissenfels and Schmelzer, 1976; Fujisawa et al., 1983; Falloon et al., 1986; Owolabi and Proll, 2000; Rabenstein et al., 2007) to AV1 as shown in **Supplementary Table S1**. They were cultivated in plastic pots containing a sand-humus mixture (3:1 v/v) and kept at 20°C–25°C in an insect-proof greenhouse. Fifteen species, belonging to four different families, were screened for their use as experimental host plants (**Supplementary Table S1**). At least three plants of each species, at six to eight leaf stages, were mechanically inoculated with 19 AV1 isolates. Those isolates selected originated from all major asparagus-growing areas distributed across Germany, resulting in samples from Baden-Württemberg, Bavaria, Brandenburg, Hesse, Lower Saxony, North Rhine-Westphalia, Rhineland-Palatinate, Saxony-Anhalt, and Schleswig-Holstein. Additionally, isolates from adjacent European countries (Netherlands, Austria) and North America as well as from import countries (Greece, Hungary, Mexico, Peru, and Spain) were included for comparison. In case of an inconsistent result, up to six plants of each species were tested. After inoculation, plants were kept in the greenhouse at 18°C–22°C with 14-h daylight. Symptom expression followed by DAS-ELISA confirmed infection with AV1. Based on macroscopic symptoms, the following disease characteristics were listed: chlorotic local lesions (C), necrotic local lesions (N), ring spot local lesions (R), leaf yellowing (Y), systemic mosaic patterns (SM), systemic necrotic lesions (SN), and no symptom (-) (compare **Supplementary Figure S1**). To differentiate local or systemic distribution of AV1, both inoculated and newly emerged leaves of each host species were tested apart. Isolates were grouped in two different pathotypes first suggested by Rabenstein et al. (2007) using the following procedure: isolates that caused only local lesions on the inoculated leaves of *Chenopodium quinoa* or *Chenopodium amaranticolor* belonged to pathotype I (PI), and isolates that caused only systemic infection in *N*. *benthamiana* belonged to pathotype II (PII).

### RNA extraction and coat protein sequences

Total nucleic acids from AV1-infected plant material were extracted according to Menzel et al. (Menzel et al., 2002). Here, 2–3 µl of heat-denatured total nucleic acid extract was used for cDNA synthesis for 60 min at 42°C with 1 µl of 10 µM primer_AV1_18_GAas: GAGATGCCATGCCGACCCTTTTTTTTTTTTTTTTTTTTTTGTCCCTTG (cDNA_1) or primer_AV1GGas1: AAAGGTCTCTACCCGTCCCTTGTTCAACACGTAC (cDNA_2), respectively, in a total volume of 20–25 µl with 2 mM dNTPs and 20–50 U RevertAid (ThermoFisher; EP0441). Subsequently, 3 µl of cDNA_1 was used in a total volume of 20 µl with Phusion Flash Master Mix directly for PCR_1 with 0.5 µM primers AV1maas3end: GAGATGCCATGCCGACCCGTCCCTTGTTCAACACGTACAATAC and AspV1_C05s: ATAGCTATTAGACCGGATCACGAG or in case of cDNA_2 for PCR_2 with 0.5 µM primers pJetGA_AV1s: GGCTCGAGTTTTTCAGCAAGATCTCGATCAAGCTCCATACAATG and pJetGA_AV1as: GTAGGAGATCTTCTAGAAAGATACTCACATAGTGCAAATGCA. PCR products were SureClean (Bioline) purified and used either for ligation into pJET (PCR_1) according to the manufacturer’s instructions or for Gibson Assembly into pJET (PCR_2) (Gibson et al., 2009). After transformation of *Escherichia coli* NM522, plasmids were purified according to a modified protocol of Birnboim and Doly (1979). Here, 1 µl of the plasmid preparation was used for amplification of the insert in a PCR using 0.5 µM primers pJs: CACCATATCCATCCGGCGTAATAC and pJas: CCTGATGAGGTGGTTAGCATAGTTC within a total volume of 20 µl Phusion Flash Master Mix. The PCR product was purified with SureClean and sequenced with primer pJs at Microsynth.

### Evolutionary analysis by maximum likelihood method

**Figure 1:**
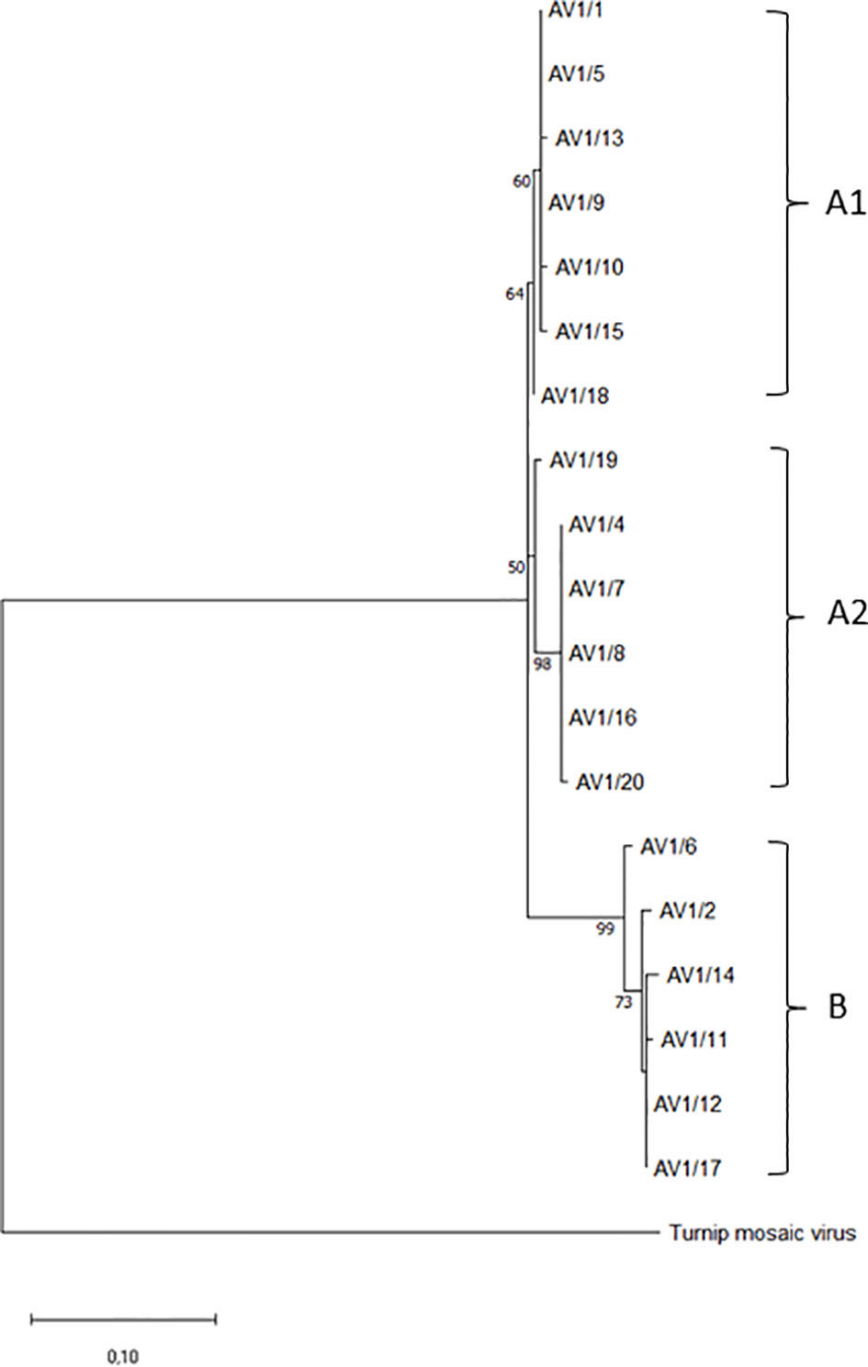
Phylogenetic tree of selected AV1 isolates from different geographic regions based on coat protein gene amino acid sequences. Two groups **(A, B)** and, within group A, two subgroups are shown. Turnip mosaic virus (TuMV, GenBank: AP017795.1) was included as an outgroup.

The sequences of the CPs were included in the GenBank database (www.ncbi.nlm.nih.gov). GenBank code numbers are given in **Table 1**. Multiple sequence alignment was performed with MUSCLE in MEGA 11 (Tamura et al., 2021). The evolutionary history was inferred by using the maximum likelihood method and JTT matrix-based model (Jones et al., 1992). The tree with the highest log likelihood (-1331.25) is shown. The percentage of trees in which the associated taxa clustered together is shown above the branches. Initial trees for the heuristic search were obtained automatically by applying Neighbor-Join and BioNJ algorithms to a matrix of pairwise distances estimated using the JTT model and then selecting the topology with superior log likelihood value. The rate variation model allowed for some sites to be evolutionarily invariable ([+I], 29.29% sites). The tree is drawn to scale, with branch lengths measured in the number of substitutions per site. This analysis involved 20 amino acid sequences and 500 bootstraps. Bootstrap values below 45% were omitted from the phylogenetic tree (**Figure 1**). All positions containing gaps and missing data were eliminated (complete deletion option). There were a total of 268 positions in the final dataset. Evolutionary analyses were conducted in MEGA11 (Tamura et al., 2021). The CP of turnip mosaic virus (TuMV, GenBank: AP017795.1) was used as an outgroup for the phylogram.

### Transmission electron microscopy

The ultrastructure of AV1 infection and adaptation to new host plants were studied using transmission electron microscopy (TEM). Single infection and purity were confirmed by detection of AV1 virions in sap homogenates of inoculated plants using negative staining and immunoelectron microscopy (IEM) with the homologous AV1 antisera (Richert-Pöggeler et al., 2018). Both natural and experimental host plants were embedded. The test plants displayed distinct susceptibilities to the potyvirus infection resulting in the following harvest dates: 42 days post-inoculation (dpi) for *A*. *officinalis*, 28 dpi for *N*. *benthamiana*, and 14 dpi for *C*. *quinoa* as well as *Chenopodium capitatum*. Samples were cut into pieces (1–2 mm²), fixed for 2 h in 2.5% glutaraldehyde and in 0.1 M phosphate buffer, and postfixed for 3 h in 0.5% osmium tetroxide and in 0.1 M phosphate buffer. The samples were kept overnight in 1% uranyl acetate in ultrapure water and dehydrated in 50% acetone, 70% acetone, and 100% acetone in each case for 30 min. Embedding was made in 1:1 acetone/EPON for 60 min at 40°C in rotator, finally placed in boxes. Polymerization occurred for 48 h at 60°C. Embedding blocks were trimmed using the TRIM2 (Leica, Vienna, Austria). Ultrathin sections (60 nm) were cut with an ultramicrotome (EM UC7 RT, Leica, Vienna, Austria), placed on nickel grids, and poststained with 2% uranyl acetate. For each sample, 10 ultrathin sections were screened. Due to technical reasons like rupture of support film on the grid, the number of cells for analyses varied from 10 to 30. Grids were examined using a TEM (Tecnai Spirit G2, FEI, Frankfurt, Germany) at 80 kV for optimal contrast. Images were taken with a 2k digital camera (Veleta, Olympus Soft Imaging Solutions, OSIS, Münster, Germany). EM images were adjusted using menus “levels,” “brightness,” “contrast,” “crop,” and “image size” of Adobe Photoshop CS4 extended.

## Results

### Broad sampling of AV1 isolates

A total of 389 asparagus samples were collected from 20 various locations, including 18 different cultivars and tested for AV1. The age of asparagus plantations was on average 3–4 years. The percentage of AV1 virus incidence was nearly 100%. Additionally, 85 asparagus spears were obtained from supermarkets. Here, the AV1 incidence varied widely: spear samples from Greece (100%), in lower levels from Peru (32%), Spain (25%), Mexico (13.3%), and Hungary (0%) (**Table 1**).

### Experimental host range studies and pathogenicity of different isolates

A total of 15 species belonging to four plant families based on previous literature data (Hein, 1960; Hein, 1969; Gröschel, 1976; Weissenfels and Schmelzer, 1976; Fujisawa et al., 1983; Falloon et al., 1986; Owolabi and Proll, 2000; Rabenstein et al., 2007) were evaluated as potential host for AV1 (**Supplementary Table S1**). Eleven species of the four families developed symptoms induced by AV1 infection (**Table 2**, **Supplementary Figure S1**). In case of the families Chenopodiaceae, Aizoaceae, and Amarantaceae, the first morphological changes were observed about 14 dpi. At 28 dpi, the *Nicotiana* species showed symptoms, while A. officinalis could not cause any symptoms at 42 dpi. The most common plant responses to AV1/1, 2, 4, 5, 6, 7, 8, 9, 10, 16, 17, 18, 19, and 20 were necrotic and chlorotic local lesions in the families Chenopodiaceae, Aizoaceae, and Amarantaceae. Additionally, the isolates AV1/2, 4, 9, 10, 11, 12, and 17 induced ring spot local lesions in *Chenopodium murale*. These symptoms occurred also in *Tetragonia expansa* (AV1/1, 2, 10, 11, 17, and 20). *Spinacea oleracea* responded to AV1/5, 17, and 20 with leaf yellowing. AV1/1, 2, 4, 5, 6, 7, 8, 9, 10, 16, 17, 18, 19, and 20 were not able to infect species in the family of Solanaceae; they were symptomless with negative ELISA results. In contrast, the isolates AV1/12, 13, 14, and 15 caused systemic infection in the species of Solanaceae and could be differentiated by symptoms shown in *Nicotiana benthamiana*, *N. clevelandii*, and *N. occidentalis*. These isolates revealed systemic mosaic infection on *N. benthamiana* and systemic necrosis on *N. clevelandii* and *N. occidentalis* on both inoculated and newly emerged leaves. While the AV1/13 and 14 exclusively showed infection in Solanaceae, AV1/12 and AV1/15 were able to infect additionally species in Chenopodiaceae like *Chenopodia capitatum*, *C. foetidum*, *C. foliosum*, and *C. murale*. Furthermore, AV1/12 showed no symptoms on C. quinoa as well as C. amaranticolor. In contrast, AV1/15 induced symptoms on C. quinoa but not on C. amaranticolor (**Table 2**). None of the tested AV1 isolates caused disease symptoms on *N. glutinosa*, *N. tabacum* ‘Samsun’, *Gomphera globosa*, and Celosia argentea or was detectable by ELISA. Based on their compatibility with indicator plants, Rabenstein et al. (Rabenstein et al., 2007) classified the AV1 isolates into two groups, referred to as pathotypes. Accordingly, PI comprised the isolates AV1/1, 2, 4, 5, 6, 7, 8, 9, 10, 11 16, 17, 18, 19, and 20 that infected only *C. quinoa* or *C. amaranticolor*, resulting in local lesions on the inoculated leaves. Isolates AV1/13 and AV1/14 causing systemic infection on *N. benthamiana* represented PII. Additionally, we could identify two isolates combining features of PI and PII isolates, AV1/12 and AV1/15 caused local lesions on *Chenopodium* spp. as well as infected *Nicotiana* spp. systemically (**Table 2**). This observation suggests the existence of a third pathotype (PIII).

**Table 2:** Symptom expression of selected indicator plant species induced by different AV1 isolates.

AV1 isolates																			
Host Species	1	2	4	5	6	7	8	9	10	11	12	13	14	15	16	17	18	19	20
Aizoceae																			
*T*. *expansa*	R	R	CN	N	CN	CN	CN	N	CR	R	–	–	–	–	CN	R	C	CN	R
Amarantaceae																			
*S*. *oleracea*	C	N	CN	Y	C	CN	C	CN	CN	C	–	–	–	–	C	Y	CN	CN	Y
*G*. *globosa*	–	–	–	–	–	–	–	–	–	–	–	–	–	–	–	–	–	–	–
*C*. *argentea*	–	–	–	–	–	–	–	–	–	–	–	–	–	–	–	–	–	–	–
Chenopodiaceae																			
*C*. *amaranticolor*	N	N	N	N	N	N	N	N	N	N	–	–	–	–	N	N	N	N	–
*C*. *capitatum*	N	N	N	N	N	N	N	N	N	N	R	–	–	N	N	N	N	N	N
*C*. *foetidum*	N	N	N	–	N	N	N	–	N	–	N	–	–	N	N	N	–	–	–
*C*. *foliosum*	N	N	N	N	N	N	N	N	N	N	N	–	–	N	N	N	N	N	N
*C*. *murale*	N	R	R	N	N	N	N	NR	R	R	R	–	–	N	N	R	N	N	N
*C*. *quinoa*	N	N	N	N	N	N	N	N	N	N	–	–	–	N	N	N	N	N	N
Solanaceae																			
*N*. *benthamiana*	–	–	–	–	–	–	–	–	–	–	SM	SM	SM	SM	–	–	–	–	–
*N*. *clevelandii*	–	–	–	–	–	–	–	–	–	–	SN	SN	SN	SN	–	–	–	–	–
*N*. *occidentalis*	–	–	–	–	–	–	–	–	–	–	SN	SN	SN	SN	–	–	–	–	–
*N*. *tabacum ‘*Samsun’	–	–	–	–	–	–	–	–	–	–	–	–	–	–	–	–	–	–	–
*N*. *glutinosa*	–	–	–	–	–	–	–	–	–	–	–	–	–	–	–	–	–	–	–

Symptom codes: C, chlorotic local lesion; N, necrotic local lesion; Y, leaf yellowing; R, ring spot local lesion; SM, systemic mosaic pattern; SN, systemic necrotic lesion; -, no infection. Inoculated and newly emerged leaves of each species were tested by DAS-ELISA. ELISA threshold value >0.1.

### Coat protein sequences and phylogenetic analysis

The phylogenetic analysis involved 20 amino acid sequences (GenBank accessions: ON548319-ON548337), including TuMV (GenBank: AP017795.1) as an outgroup. The sequences contained a total of 268 amino acid positions. Two main groups (A, B) were formed out of the sequences of the amino acids of the CP gene, with bootstrap support of 64% for A and 99% for B. Group A has two subgroups (A1, A2), with bootstrap support of 60% and 50%, respectively (**Figure 1**). Isolates from Saxony-Anhalt (AV1/1 and 5) and the Netherlands (AV1/13 and 18) were in group A1, which also contained isolates from Bavaria (AV1/9), North Rhine-Westphalia (AV1/10), and Schleswig-Holstein (AV1/15). The isolates (AV1/19, 4, 7, 8, and 16) from group A2 were all from Germany, except one isolate from the United States (AV1/20). Isolates from Greece (AV1/6), Saxony-Anhalt (AV1/2), Brandenburg (AV1/14), PV-0955 (AV1/11), PV-0954 (AV1/12), and Austria (AV1/17) were classified into group B.

### Ultrastructural changes associated with AV1 infection in asparagus and in indicator plants

AV1 virions consist of flexuous filamentous particles. Serologically, all AV1 isolates reacted with the AV1 antibody as illustrated by IEM (**Supplementary Figure S2**). All selected plant samples tested were positive for AV1 infection using IEM. Embeded tissues of these plants contained typical potyviral inclusion bodies (ibs) (**Table 3**) formed by the nonstructural viral CI protein. Depending on their orientation in the cell, they appeared as pinwheels or scrolls when cross sectioned or as bundles when longitudinally sectioned or laminated aggregates.

**Table 3:** Ultrastructural changes in asparagus and different host species through AV1 pathotypes.

Host species	*A*. *officinalis* (42 dpi)		*Chenopodium* spp. (14 dpi)		*N*. *benthamiana* (28 dpi)		
Pathotype	PI	PIII	PI	PIII	PII	PIII	
**Isolate**	AV1/11	AV1/12	AV1/11	AV1/15	AV1/13	AV1/12	AV1/15
Ultrastructural changes							
Pinwheels	+	+	++	+++	+++	++	+++
Laminated aggregates	–	–	+	++	–	+	+
Scrolls	–	–	+	+++	++	+	+++
Amorphous electron-dense inclusion bodies	–	+	–	–	+	+	–
Bundles	+	++*	++	+++*	++	++	++
Mitochondria	–	–	swollen	–	elongated	–	–
Single-membrane vesicles	+	–	–	–	–	+	+
Chloroplast	–	degraded	–	–	–	–	degraded
Plastoglobuli	–	+	–	+++	+	–	++
Stromules	–	+	–	–	–	–	–

*Bundles close to cell wall; -, not changed/not present; +, single/less; ++, increased; +++, abundant. Ten ultrathin sections were screened for each plant, and between 10 and 30 cells for each plant were analyzed.

**Figure 2:**
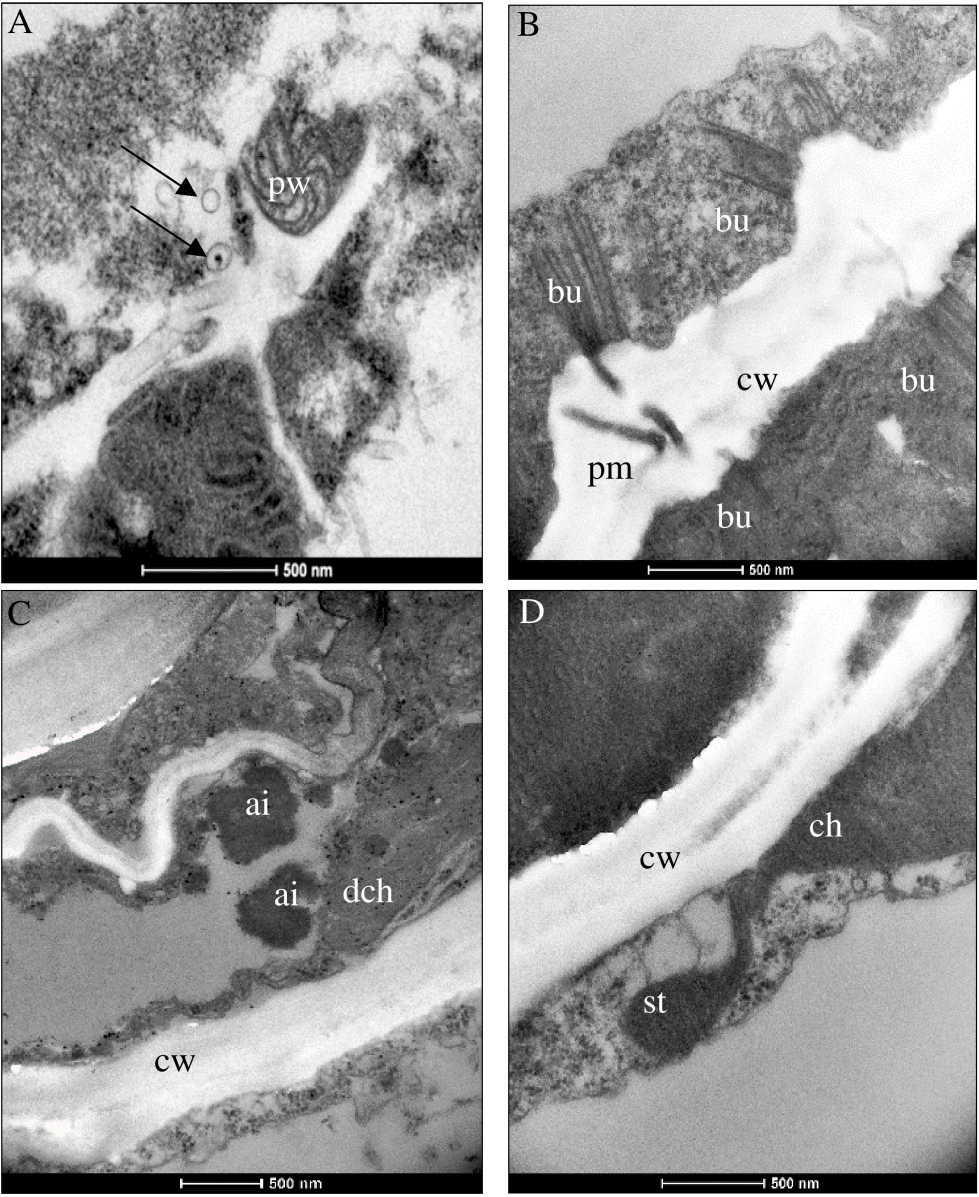
Ultrastructural changes in **(A)** *Asparagus officinalis* infected with distinct pathotypes of AV1 isolates at 42 dpi. Pathotype I (AV1/11) depicted in panel **(A)** and pathotype III (AV1/12) in panels **(B–D)**. **(A)** Pinwheels (pw) and single-membrane vesicles (arrows). **(B)** Bundles (bu) at the cell wall (cw) next to the plasmodesmata aperture (pm) connecting two cells. **(C)** Amorphous electron-dense inclusion bodies (ai) next to a degraded chloroplast (dch) and plastoglobuli (arrows). **(D)** Stromule (st) extended from ch.

Virus replication occurred in asparagus and in indicator plants, as summerized in **Table 3**. Despite the lack of symptoms, significant ultracellular changes in asparagus, the phylloclade tissue, as well as in the stem tissue came up in AV1-infected cells. These consisted of pinwheels flanked by single-membrane vesicles (**Figure 2A**). Bundles localized perpendicular to the cell wall and seemed to be connected with plasmodesmata (**Figure 2B**). At these positions, the cell wall was increased in width and amorphous inclusion bodies localized next to the chloroplast in case of PIII (**Figure 2C**). In one instance, a chloroplast formed a stromule (**Figure 2D**).

**Figure 3:**
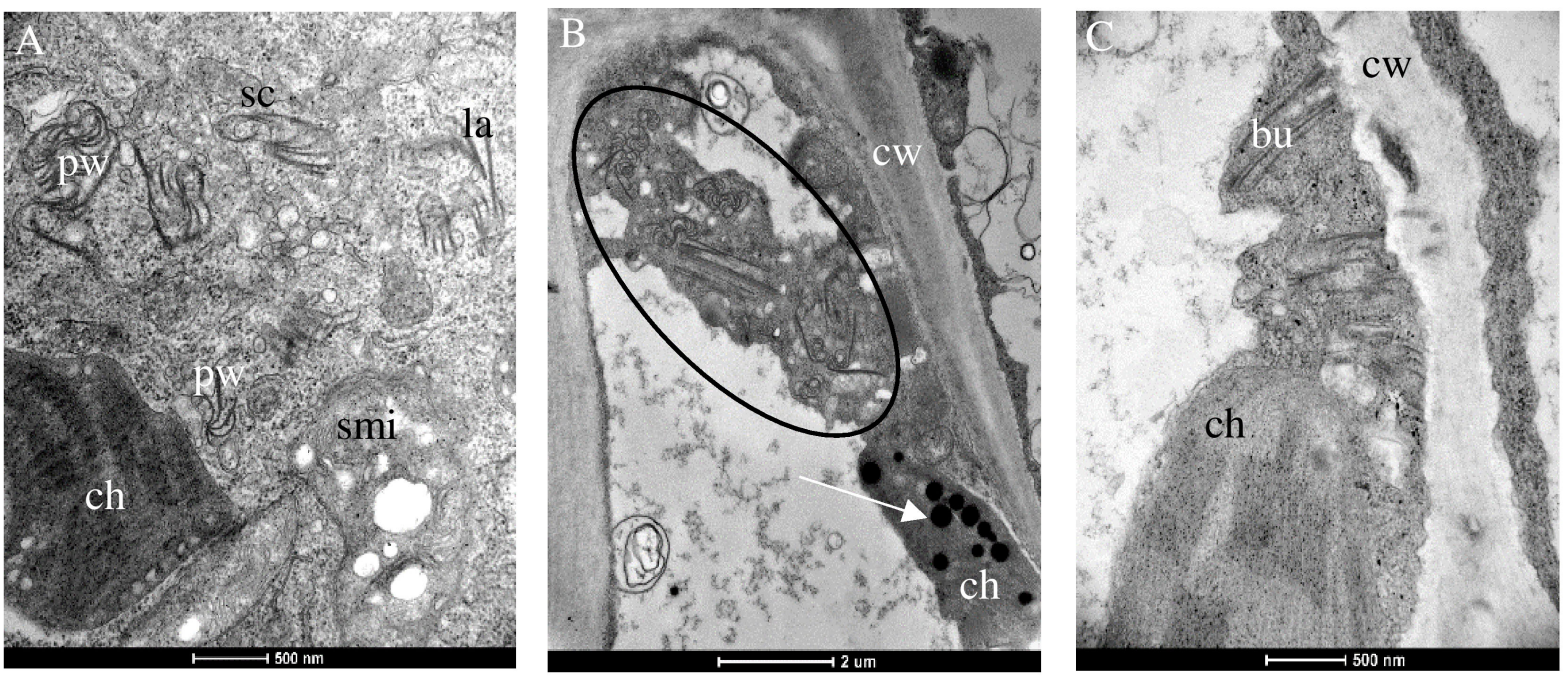
Changes in the cellular ultrastructure formed during infection of the local lesion host *Chenopodium* spp. by pathotypes I **(A)** and III **(B)**, respectively. **(A)** Different shapes of the CI protein consisting of Pinwheels (pw), scrolls (sc), laminated aggregates (la), and swollen mitochondria (smi) are visible in *Chenopodium quinoa* infected with PI. **(B)** Different shapes of the CI protein consisting of pw, sc, la (circle), and ch containing plastoglobuli (arrow) in **(C)** *C. capitatum* infected with PIII (AV1/15). **(C)** Bu next to the cell wall (cw).

*Chenopodium* species display multivirus resistance. PII was not able to overcome the host resistance, and infection was not established after inoculation (**Table 2**). Accordingly, only AV1 isolates of PI and PIII that triggered a hypersensitive resistance in the bioassay (**Table 2**) were selected for ultrastructural analysis of the infected tissues. None of these pathotypes infected the *Chenopodium* spp. systemically. Only inoculated leaves showed local lesions as symptoms (**Table 2**). PI (isolate AV1/11) produced pinwheels, laminated aggregates, and scrolls in the cytoplasm of infected tissue, and the mitochondria appeared swollen (**Figure 3A**). A similar diversity in CI protein-derived shapes existed in PIII-infected tissues (AV1/15, **Table 3**, **Figure 3B**). Bundles occurred in proximity or close to the cell wall (**Figures 3B, C****)**. In PIII-infected tissue, chloroplasts contained several plastoglobuli (**Figure 3B**).

**Figure 4:**
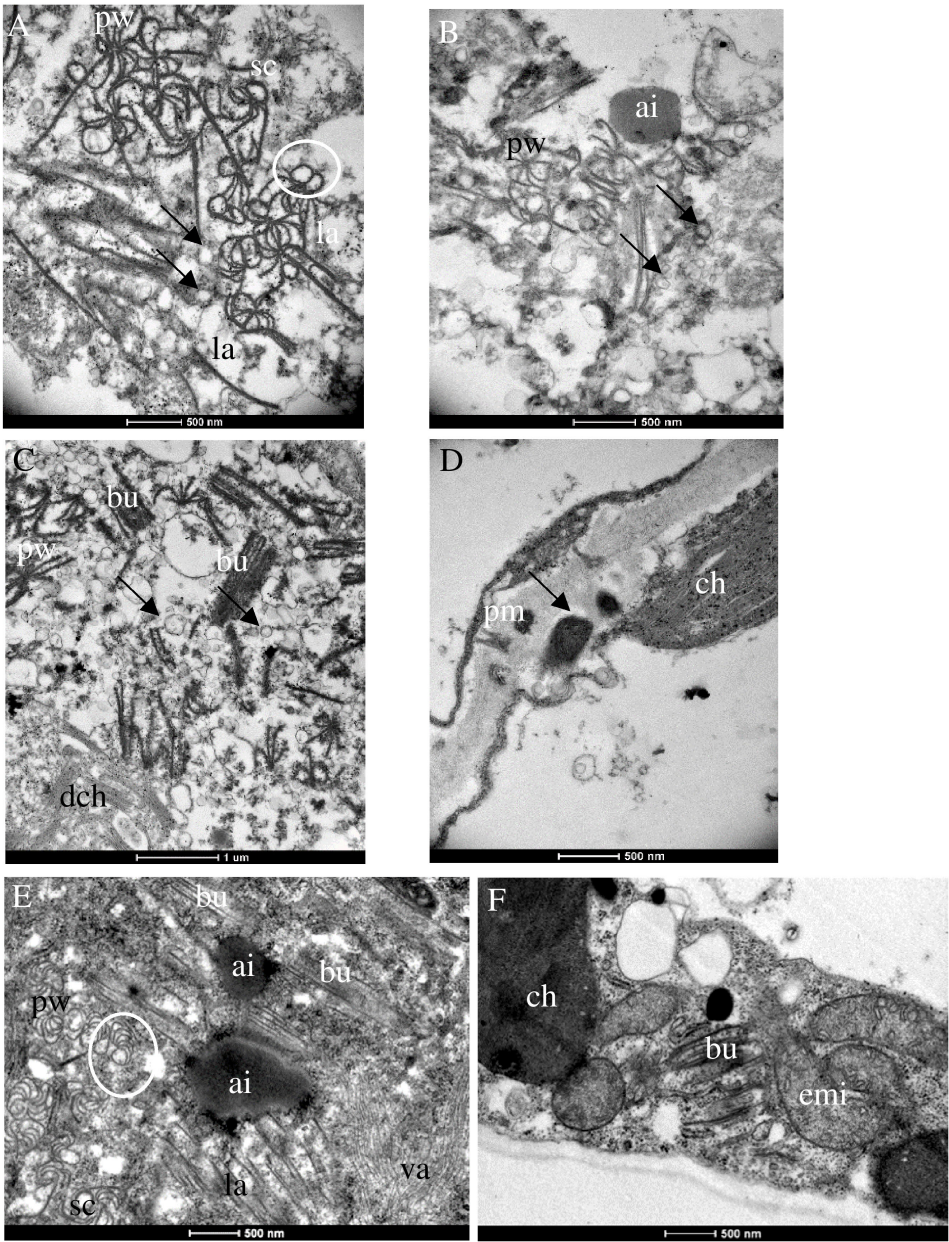
Ultrastructural changes in *N. benthamiana* infected with PIII (AV1/12, 15) **(A–D)** and PII (AV1/13) **(E, F)**. **(A)** Pinwheels (pw), sc, and laminated aggregates (la) associated with single-membrane vesicles (arrows). **(B)** Amorphous inclusion body (ai) next to pw. **(C)** Pw, bu, associated with single-membrane vesicles (arrows) and released thylakoids by degraded ch (dch). **(D)** Arrow indicates fibrillar structures flanked by plasmodesmata. **(E)** Pw, scrolls (sc), Bundles (bu), and la surrounding an ai. Virion aggregates (va) in the lower right part. **(F)** Elongated mitochondria (emi) and bu. White circles indicate sc generated by rounding of pw arms.

The effects of PII (isolate AV1/13) and of PIII (isolates AV1/12 and AV1/15) on the cellular ultrastructure of systemically infected *N. benthamiana* were compared in symptomatic tissues 28 dpi. Multiple CI protein-derived ibs and virion aggregates were found in the cytoplasm of mesophyll cells that associated with single-membrane vesicles (**Figures 4A–D**). In both pathotypes, pinwheels transformed into scrolls by rounding of the pinwheel arms (**Figure 4A**). Additional to multiple shapes of CI protein-generated aggregates, amorphous inclusion bodies formed in both infections (**Figures 4B, E****)**. Chloroplasts degraded and released their content in the cytoplasm (**Figures 4C, F****)**. Increased production of plastoglobuli was seen in the chloroplast (**Supplementary Figure S2**). The cell wall appeared expanded due to electron-dense material with fibrillar structures flanked by plasmodesmata (**Figure 4D**). In PII-infected cells, the mitochondria became elongated (**Figure 4F**).

## Discussion

This work describes the properties of 19 AV1 isolates based on experimental host range, pathogenicity, phylogeny of the CP, and alterations in the ultrastructure of infected cells. Our monitoring showed a wide distribution of AV1 in large asparagus production regions in the world. Asparagus samples from German asparagus-growing regions and selected European and American countries showed a nearly 100% infection rate(**Table 1**), confirming data from previous reports (Hein, 1960; Hein, 1969; Weissenfels and Schmelzer, 1976; Montasser and Davis, 1987; Kegler et al., 1991; Bandte et al., 2008; Nothnagel et al., 2011). There are multiple reasons for the observed infection. In all cases, the plant age of the collected samples was higher than 2 years, allowing virus spread within the field by aphids. Increasing age of asparagus plantations plays an important role in the increase of AV1 infection, as demonstrated by Weissenfels and Schmelzer (Weissenfels and Schmelzer, 1976). While the infection rate was 21% in the 1-year-old plantation, it was 58% in the 2-year-old plantation and 73% in the 3-year-old one. The virus is mainly spread by the green peach aphid *M. persicae* in a nonpersistent manner (Fujisawa et al., 1983). This mode of transmission is extremely rapid, so control with insecticides is not feasible because by the time the aphid is killed, the virus has already spread to another plant. We have also tested asparagus spears from Greece, Hungary, Spain, Peru, and Mexico on AV1. The values obtained here ranged from 0% to 100% (**Table 1**). The AV1 infection of asparagus spears from Peru and Mexico was lower compared with that of North America and Germany, which may be due to the location of asparagus-growing areas close to the Pacific coast, where aphid flight is limited. Another explanation might be strict decontamination of knives during the harvest of asparagus. Mechanical transmission of the virus by contaminated knives was demonstrated by Kegler et al. (Kegler et al., 1991). The sample numbers from Greece, Hungary, and Spain were very small and do not allow any conclusions to be drawn about the national AV1 incidence. Systemic virus spread of AV1 within asparagus leads to reduced yields of spears and negatively affects, among others, the brix values, which were shown to decrease in greenhouse experiments for an AV1 isolate from Germany (Lantos et al., 2018). In our survey, we obtained 19 isolates comprising different geographic origins worldwide to identify the diversity of AV1. It was rather difficult to recognize the pathogenicity of isolates using phylloclades. The cylindrical anatomy of asparagus phylloclades (Nakayama et al., 2012) letting them appear like stems rather than leaves prevents easy recognition of virus-specific symptoms such as chlorotic mottling or mosaic or necrosis (Hein, 1969; Weissenfels and Schmelzer, 1976). Therefore, we carried out a host range study including 15 herbaceous species from four different plant families (**Supplementary Table S1**). The multivirus resistance of *Chenopodium* spp. included AV1 and made them useful local lesion hosts to monitor the pathogenicity of most AV1 isolates, as shown in our study as well as by others (Hein, 1960; Hein, 1969; Gröschel, 1976; Weissenfels and Schmelzer, 1976; Fujisawa et al., 1983; Falloon et al., 1986; Cooper, 2001). The identification of novel isolates in our study may explain the difference in observations reported from other authors on AV1 susceptibility/resistance when *T*. *expansa*, *S*. *oleraceae*, *G*. *globosa*, *C*. *argentea*, or *Nicotiana* spp. were used in bioassays (Hein, 1960; Hein, 1969; Gröschel, 1976; Fujisawa et al., 1983; Owolabi and Proll, 2000; Rabenstein et al., 2007). AV1 pathotypes have been defined by their ability to infect either *Chenopodium* spp. or *N. benthamiana* (Rabenstein et al., 2007). Accordingly, 15 isolates were classified as PI and two isolates were grouped as PII. Interestingly, two isolates infected both *Chenopodium* spp. and *Nicotiana* spp. and therefore we propose the existence of a third pathotype PIII. Furthermore, the host plant study revealed aspects of adaptation of AV1 to dicotyle hosts and the interference of AV1 with different layers in the plant defense system (Moury and Desbiez, 2020). The host range of AV1 can be even extended to plant orders like Caryophyllales and Solanales that are phylogenetically distant from Asparagales (The Angiosperm Phylogeny Group et al., 2016). It is interesting to note that PII is capable of inducing a hypersensitive resistance (HR) when inoculated on *Chenopodium* spp. (Caryophyllales) but does neither trigger HR or an infection when experimentally transmitted to *N. glutinosa and N. tabacum* ‘Samsun’ (Solanales) [this study, 8,37,38]. Apparently, AV1 proteins are not recognized as effector molecule by the N gene, a nucleotide-binding site leucine-rich repeats (TIR-NB-LRR) protein (Huang, 2021). In contrast to former reports on local infections by AV1, the described isolates of PII and PIII seem to escape the HR response by the host partly in *N. clevelandii* and *N. occidentalis* and are able to reach the phloem allowing systemic infection.

The CP of potyviruses serves not only for the recognition and the packaging of the viral genome but acts also as a regulatory element in virus replication as well as elicitor of effector-triggered immunity (Martínez-Turiño and García, 2020). We therefore did a phylogenetic analysis of the CP from the AV1 isolates at the amino acid level to estimate its contribution to the isolates’ diversity. Neither the two major clusters A and B nor the subclusters A1 and A2 within the phylogenetic tree did match with geographical or CI grouping of pathotypes. In addition, no correlation was found between these groups and a symptomatic phenotype or the host plant spectrum. Hence, the CP does not appear to be solely responsible for, e.g., systemic infection in *Nicotiana* spp. or local lesion formation in *Chenopodium* spp. Whole-genome sequencing and bioinformatics will be useful tools to understand pathotype evolution and identify possible recombination hot spots within the AV1 genome as well as differences in sequence or structure of virulence factors such as CI.

As mentioned earlier, AV1-infected asparagus does not show visible symptoms despite the fact that the virus spreads systemically in the plant. The successful transmission of selected AV1 isolates to asparagus as well as to indicator plants allowed for the first time comparisons of their histopathology in response to the various pathotypes (**Figures 2**–**4**). It revealed changes in the cellular ultrastructure triggered by pathotypes that showed distinct virulence in the bioassay (**Table 2**). AV1 infection generated cellular changes, like inclusion bodies (ibs) typical for potyvirus infection as already have been reported in *C. quinoa* (Gröschel and Jank-Ladwig, 1977; Howell and Mink, 1985) and in C. amaranticolor (Fujisawa et al., 1983; Marani et al., 1993). Until now, there are only two studies available describing cellular changes in asparagus by one isolate of AV1 infection. The development of pinwheels, laminated aggregates, and bundles was observed in the cell infected with AV1 in addition to the appearance of vesicular structures (Fujisawa et al., 1983). The study of Marani et al. (1993) described structural changes like deformed chloroplasts and abnormal mitochondria in asparagus. In our study, we observed that in asparagus, the appearance of pinwheels and scrolls was reduced independent of both pathotypes PI and PIII (**Table 3**, **Figure 2A**, **Supplementary Figure S3**). In contrast, the same pathotypes caused more ibs per cell in *Chenopodium* spp. (**Figures 3A–C**). The most ibs per cell by PII and PIII were shown in *N. benthamiana* (**Figures 4A–C, E**). Bundles were in each host detectable independent of pathotypes. However, there was one difference in the location of bundles between pathotypes and host. Bundles of PIII in asparagus were perpendicular to the cell wall and were observed at both ends of the plasmodesmata, indicating cell-to-cell spread of AV1 (**Figure 2B**). This observation of bundles has not been previously reported by AV1 infection. These bundles are formed through CI protein, which are involved in cell-to-cell movement and systemic spread of the virus (Sorel et al., 2014). PIII induced also bundles in infected cell of *C. capitatum*, however, only at one side of the cell wall (**Figure 3C**), indicating hypersensitive resistance to AV1 (Cooper, 2001). Chloroplasts of AV1 (PIII)-infected cells were degraded in asparagus as well as in *N. benthamiana* (**Figures 2C**, **4C**). In addition, the appearance of stromule in asparagus by PIII was also noted, as shown in **Figure 2D**. A stromule is a tubular projection that can develop from chloroplasts as a result of viral defense (Caplan et al., 2015). Furthermore, in each host, an increased number and size of plastoglobuli were seen, which may affect negatively photosynthesis as described by Reinero and Beachy (1989) and Díaz-Vivancos et al. (2008) (**Figures 2C**, **3B**; **Supplementary Figure S4**). Membrane-less amorphous inclusion bodies that were only reported from some potyvirus infections (Zielińska et al., 2012) were observed for PII and PIII (**Figures 2C**, **4E**). We were not able to distinguish their ultrastructure due to their electron density. Single-membrane vesicles in asparagus (**Figure 2A**) and *N. benthamiana* (**Figures 4B, C**) work as replication organelles, which can be formed by virus by rearranging the cell membrane of the host to facilitate viral replication (Nguyen-Dinh and Herker, 2021).

Until now, there are no AV1-resistant cultivars of garden asparagus available. In contrast, resistance to AV1 PI (AV1/1 ON548319) could be attested in various asparagus wild relatives (Nothnagel et al., 2017). Meanwhile, an introgression breeding program has been initiated and showed that these resistances can be transmitted to garden asparagus (Plath et al., 2018). Recently, the successful transfer of the AV-1^pro^ gene from *Asparagus prostratus* to chromosome 2 of garden asparagus was reported (Nothnagel et al., 2022). The durability of the resistance and effectiveness for other AV1 pathotypes will be the subject of further research. The recent work demonstrates significant variability among the 19 AV1 isolates reflected by three different pathotypes. In addition to the AV-1^pro^ resistance, the resistance donors of other wild species, e.g., *A. amarus*, *A. maritimus*, or *A. pseudoscaber*, can therefore also be important for the generation of a durable resistance in garden asparagus.

## Data availability statement

The datasets presented in this study can be found in online repositories. The names of the repository/repositories and accession number(s) can be found below: https://www.ncbi.nlm.nih.gov/genbank/, ON548319-ON548337.

## Author contributions

EL contributed to the paper conception and experimental approaches. RK contributed to the paper conception. EM contributed to the RNA and phylogenetic approach. KR-P contributed to the transmission electron microscopy. JK contributed to the AV1 test approach. TN contributed to the project initiation, plant development, and paper conception.
