# Supplementary material for: Host range and molecular and ultrastructural analyses of Asparagus virus 1 pathotypes isolated from garden asparagus Asparagus officinalis L
Source: Front Plant Sci. 2023 Jul 31;14:1187563. doi: 10.3389/fpls.2023.1187563 (PMC10433173; doi:10.3389/fpls.2023.1187563)

FIGURE S1

The most common symptoms caused by AV1.

A. Local symptoms

Ring spot local lesions on *Tetragonia expansa* (left) and *Chenopodium murale* (right)

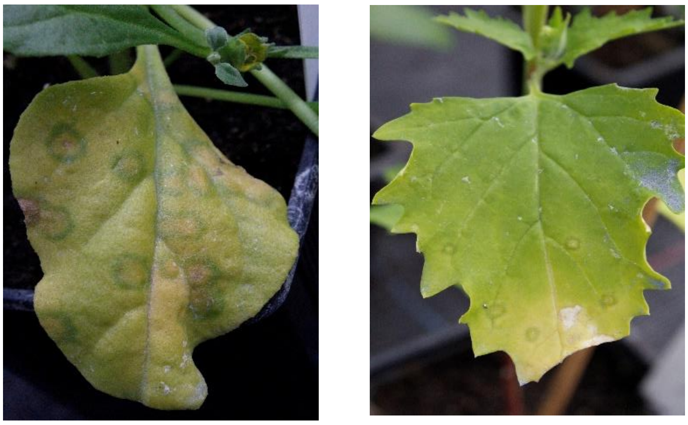

Necrotic local lesions on *Chenopodium foetidum* (left) and *Chenopodium capitatum* (right)

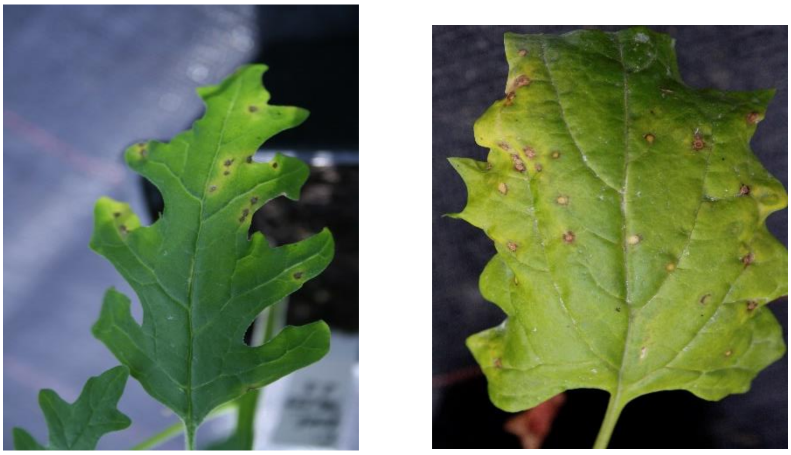

Chlorotic local lesion on *Spinacea oleraceae*

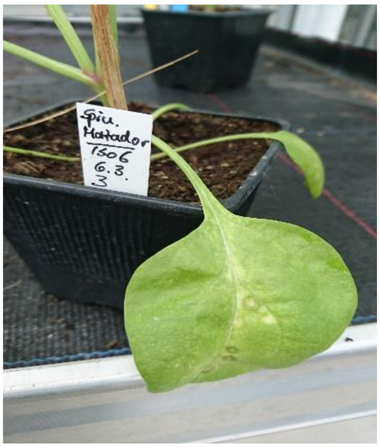

B. Systemic symptoms

Systemic mosaic pattern on *Nicotiana benthamiana*

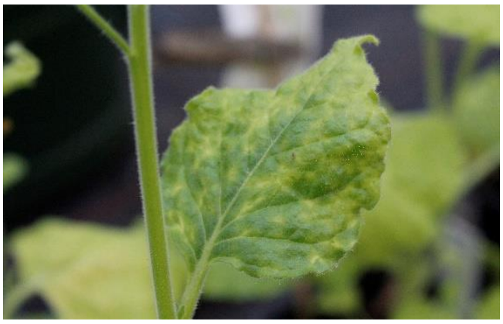

Systemic necrotic lesions on *Nicotiana occidentalis* (left) and *Nicotiana clevelandii* (right)

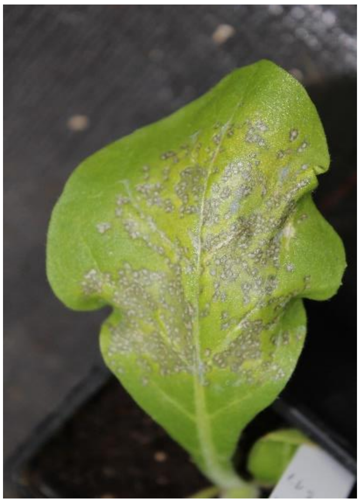

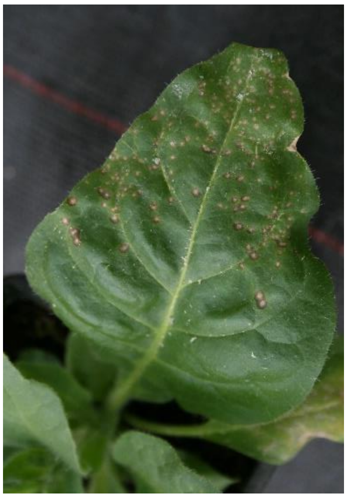

Supplement: Supplementary file 1 [file Image_1.pdf]
