# Supplementary figures and images for: Host range and molecular and ultrastructural analyses of Asparagus virus 1 pathotypes isolated from garden asparagus Asparagus officinalis L
Source: Front Plant Sci. 2023 Jul 31;14:1187563. doi: 10.3389/fpls.2023.1187563 (PMC10433173; doi:10.3389/fpls.2023.1187563)

FIGURE S2

AV1 virion decorated (*C. quinoa*, AV1 isolate 11).

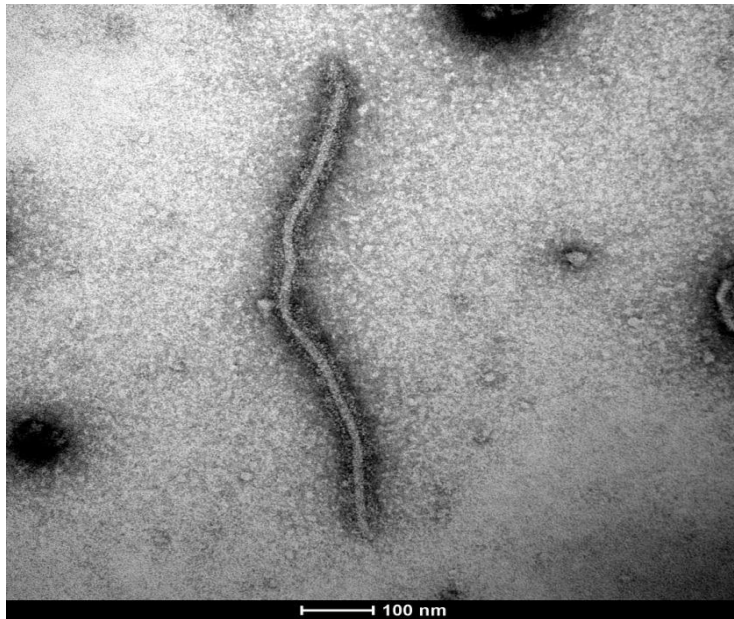

Supplement: Supplementary file 2 [file Image_2.pdf]

FIGURE S3

Pinwheels (arrow) in asparagus (AV1/12).

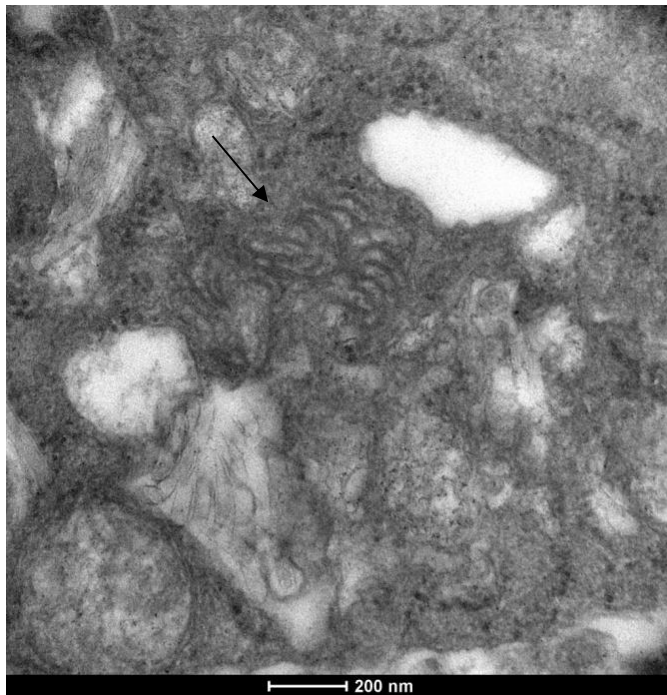

Supplement: Supplementary file 3 [file Image_3.pdf]

FIGURE S4

AV1 infected chloroplast showed plastoglobuli (arrows) in *N. benthamiana* (PIII).

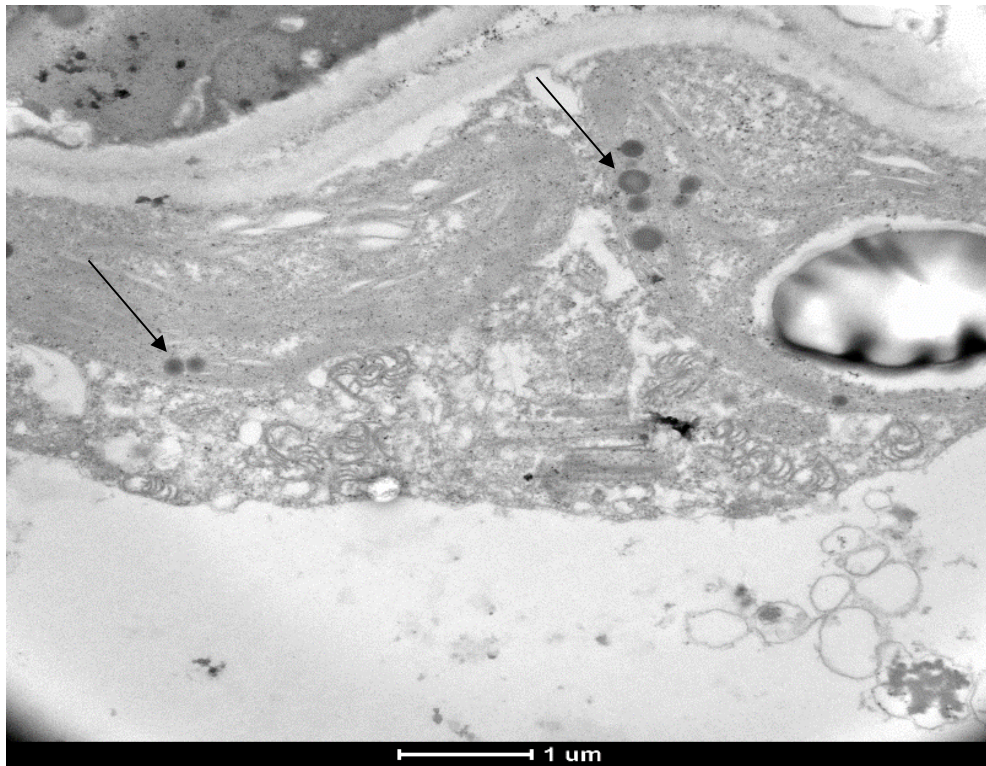

Supplement: Supplementary file 4 [file Image_4.pdf]
