# Supplementary material for: Host range and molecular and ultrastructural analyses of Asparagus virus 1 pathotypes isolated from garden asparagus Asparagus officinalis L
Source: Front Plant Sci. 2023 Jul 31;14:1187563. doi: 10.3389/fpls.2023.1187563 (PMC10433173; doi:10.3389/fpls.2023.1187563)
Supplement: Supplementary file 5 [file Table_1.docx]

Supplementary Material

Host Range, Molecular and Ultrastructural Analyses of asparagus virus 1 pathotypes Isolated from Garden Asparagus *Asparagus officinalis* L.

Edit Lantos, Reiner Krämer , Katja R. Richert-Pöggeler , Edgar Maiß , Janine König, Thomas Nothnagel *

*** Correspondence:** Corresponding Author: [thomas.nothnagel@julius-kuehn.de](mailto:thomas.nothnagel@julius-kuehn.de)

**Table S1.** List of plant species used in experimental host range study.

| **Family** | **Species** | **Literature data** |
| --- | --- | --- |
| Aizoaceae | *Tetragonia expansa* L. | Gröschel 1976; Fujisawa et al. 1983 |
| Amaranthaceae | *Spinacea oleracea* L. | Hein 1969; Gröschel 1976; Fujisawa et al. 1983 |
|  | *Gomphrena globosa* L. | Hein, 1960; Hein 1969; Weissenfels and Schmelzer 1976; Mink and Uyeda 1977  Gröschel 1976; Fujisawa et al. 1983; Falloon and Falloon 1986; Owolabi and Proll 2000 |
|  | *Celosia argentea* L. | Fujisawa et al. 1983; Owolabi and Proll 2000 |
| Chenopodiaceae | *Chenopodium amaranticolor* L. | Weissenfels and Schmelzer 1976; Mink and Uyeda 1977; Fujisawa et al. 1983; Falloon and Falloon 1986; Owolabi and Proll 2000, Rabenstein et al. 2007 |
|  | *Chenopodium capitatum* L. | Weissenfels and Schmelzer 1976; Gröschel 1976; Fujisawa 1983; Falloon and Falloon 1986; |
|  | *Chenopodium foetidum* C. Schrad. | Weissenfels and Schmelzer 1976; Gröschel 1976; |
|  | *Chenopodium foliosum* L. | Hein 1969; Weissenfels and Schmelzer 1976; |
|  | *Chenopodium murale* L. | Weissenfels and Schmelzer 1976; Gröschel 1976; Owolabi and Proll 2000; |
|  | *Chenopodium quinoa* Willd. | Hein 1960; Hein 1969; Weissenfels and Schmelzer 1976; Gröschel 1976; Mink and Uyeda 1977; Fujisawa et al. 1983; Falloon and Falloon 1986; Owolabi and Proll 2000; Rabenstein et al. 2007 |
| Solanaceae | *Nicotiana benthamiana* L. | Fujisawa et al. 1983; Owolabi and Proll 2000; Rabenstein et al. 2007 |
|  | *Nicotiana clevelandii* A. Gray | Gröschel 1976; Fujisawa et al. 1983; Owolabi and Proll 2000, Tomassoli et al. 2008a |
|  | *Nicotiana glutinosa* L. | Hein 1969; Gröschel 1976; Fujisawa et al. 1983; |
|  | *Nicotiana occidentalis* H.-M. Wheeler | Owolabi and Proll 2000; |
|  | *Nicotiana tabacum* L. “Samsun” | Hein 1969; Gröschel 1976; Fujisawa et al. 1983 |
